# Recent Increase in Methamphetamine Use in a Cohort of Rural People Who Use Drugs: Further Evidence for the Emergence of Twin Epidemics

**DOI:** 10.3389/fpsyt.2021.805002

**Published:** 2022-01-07

**Authors:** Jennifer R. Havens, Hannah K. Knudsen, Justin C. Strickland, April M. Young, Shanna Babalonis, Michelle R. Lofwall, Sharon L. Walsh

**Affiliations:** ^1^Department of Behavioral Science, Center on Drug and Alcohol Research, University of Kentucky College of Medicine, Lexington, KY, United States; ^2^Department of Psychiatry and Behavioral Sciences, Johns Hopkins University School of Medicine, Baltimore, MD, United States; ^3^Department of Epidemiology, University of Kentucky College of Public Health, Lexington, KY, United States

**Keywords:** methamphetamine, rural, Appalachian Kentucky, epidemic, opioid

## Abstract

Appalachian Kentucky was at the epicenter of the prescription opioid epidemic in the early 2000's. As we enter the third decade of the epidemic, patterns have begun to emerge as people who use drugs (PWUD) transition from use of opioids to other drugs. The purpose of this analysis was to examine longitudinal changes in methamphetamine use in an ongoing cohort of rural people who use drugs (PWUD) in Appalachian Kentucky. All but five of the cohort participants (*N* = 503) reported nonmedical prescription opioid use (NMPOU) at baseline and those 498 are included in this longitudinal analysis encompassing eight waves of data (2008–2020). Past 6-month use of methamphetamine was the dependent variable. Given the correlated nature of the data, mixed effects logistic regression was utilized to examine changes in methamphetamine use over time. Significant increases in methamphetamine use were observed over the past decade in this cohort of PWUD, especially in recent years (2017–2020). Prevalence of recent use at baseline and each of the follow-up visits was as follows: 9.4, 5.6, 5.0, 5.4, 8.1, 6.8, 6.9, and 33.1%, respectively (*p* < 0.001). On the contrary, significant reductions in NMPO and heroin use were observed in the same time period. The odds of methamphetamine use at the most recent visit were 25.8 times greater than at baseline (95% CI: 14.9, 44.6) and 52.6% of those reporting methamphetamine use reported injecting the drug. These results provide further evidence of “twin epidemics” of methamphetamine use among NMPOU. While problematic on several fronts, of particular concern is the lack of effective treatment options for methamphetamine use disorder. As policies around the opioid epidemic continue to evolve, particular attention should be paid to the surge in stimulant use in opioid-endemic areas.

## Introduction

The opioid epidemic has been well documented in the United States (1, 2). However, there is still uncertainty around how the epidemic will progress. The first major shift after recognition of a prescription opioid epidemic was the transition from nonmedical prescription opioids (NMPO) to heroin use (3). While somewhat expected given the pharmacologic similarities between prescription opioids and heroin (4), this transition remains concerning due to risk of overdose (5, 6), contamination of heroin supplies with fentanyl and fentanyl-analogs and its related harms (7, 8), and a dearth of harm reduction services in many areas of the U.S. to combat heroin- and opioid-related issues (9). Recent data suggest that we may be entering yet another new era of the opioid epidemic, where those using NMPO and/or heroin begin concomitant use of methamphetamine (10–14). Coined “twin epidemics” (13), this phenomenon has now been studied in substance use disorder (SUD) treatment samples (10, 11, 13), a cross-sectional study of mid-western NMPOU's (14) and nationally-representative samples (12, 15), but has not been studied longitudinally among those using opioids. Increased methamphetamine use raises considerable concern as it is associated with a litany of harms; including, among others, dental issues (16, 17), cardiac abnormalities (18, 19), and transmission of infectious diseases, such as HIV and hepatitis C *via* sharing of infected pipes and injection implements, as well as engagement in risky sex (20–23). Methamphetamine use is not novel, especially in rural areas of the U.S. (24, 25) and among those using drugs to enhance sex (“chemsex”) (26, 27); however, there is growing body of evidence that use is increasing in new populations of established people who use drugs (PWUD), and people using opioids in particular (12, 14).

The emergence of methamphetamine use among people using opioids is particularly problematic given the lack of effective treatment options for methamphetamine use disorder (MUD), especially compared to opioid use disorder (OUD). While there are several medications currently under study, no FDA-approved pharmacologic treatments exist for MUD (28, 29). A 2017 systematic review of the evidence-based treatment options identified several behavioral interventions, including cognitive behavioral therapy (CBT), motivational interviewing (MI), and contingency management (CM), among others (30). Of those, CM appeared to be most efficacious in reducing methamphetamine use in the short-run, along with CBT and exercise, in certain settings (30). A more recent overview of published systematic reviews noted significant reductions in amphetamine use when psychsocial interventions are employed (31). However, rural areas in particular may be ill-equipped to deliver interventions requiring skilled mental health providers that are often in short supply (32) and CM, while very promising, is not a reimbursable treatment because giving incentives is equated to a “kick-back” and considered unlawful by many insurers, including Medicaid (33).

Although Europe has been largely spared from a NMPO epidemic, data indicate that European countries may not be entirely immune (34, 35). There have been several reports of increasing prescribing of opioids in the Netherlands (36, 37), UK (38), Sweden (39), and France (40), which may be a signal for problematic NMPOU. A 2017 study comparing the use of opioids in the U.S. and Europe suggests troubling patterns of opioid use in the United Kingdom that mirror the U.S. (35). Reports from Australia also indicate that opioid prescribing has increased in recent years (41–43), as have concerns about the potential for NMPOU (43). Another potential signal of problematic opioid use in Australia was overdose data showing the proportion of fatal overdoses where prescription opioids were present was 2.5 times that of heroin (44). Even though the U.S. opioid epidemic is ever-evolving, what has transpired thus far may inform the response in countries where the potential for NMPOU use has increased in recent years. It is therefore important to examine long-term outcomes of the opioid epidemic, especially in cohort studies largely comprised of NMPOUs. The aims of these analyses were to examine changes in methamphetamine use over time and explore characteristics of those individuals using methamphetamine within a cohort of rural people who use opioids followed from 2008–2020.

## Materials and Methods

Data from the Social Networks among Appalachian People (SNAP) study were utilized for the current analysis. At baseline the cohort consisted of 503 community-dwelling residents of a rural county in Appalachian Kentucky. Those eligible for the SNAP study reported past 30-day use of either NMPO, cocaine, methamphetamine or heroin. An extensive description of the methods for the SNAP study are provided elsewhere (45). Of note, all but five participants reported recent (past 6-month) NMPOU at baseline, and all 503 participants reported lifetime NMPOU. Those indicating recent NMPO use at baseline (99%) are included in the current analysis (*N* = 498). Participants were remunerated $50 at each visit. The study was approved by the Institutional Review Board at the University of Kentucky and a Certificate of Confidentiality was obtained from the National Institutes of Health.

Data were collected bi-annually for the first wave of the study (2008–2013), and annually thereafter (2014–2020) for a total of eight study visits. Follow-up rates were 92.3, 92, 93.7, 90, 89.1, 89.7, and 83.9% for the 1st–7th follow-up visits, respectively. The survey was approximately 90 min in length and interviewer-administered. Responses were recorded directly on to a touchscreen laptop using computer-assisted personal interviewing (CAPI) software (QDS, Bethesda, MD).

### Study Variables

Data from the baseline and seven follow-up visits were utilized for the longitudinal trend analysis (*n* = 498) and data from the most recently completed follow-up visit (*n* = 350) were utilized to characterize methamphetamine use in this sample of rural people who use opioids. The dependent variable of interest was recent (past 6-month) methamphetamine use at baseline and each follow-up visit. To ascertain whether participants had used methamphetamine, they were asked “Have you ever used methamphetamine” and if so, “How often have you used methamphetamine in the past 6 months”? The second question was dichotomized to include those with any/no use to create the recent use variable that was used as the dependent in all analyses. Other substance use was assessed contemporaneously with methamphetamine use and recent use variables were created for each substance analyzed (NMPO, heroin, benzodiazepines, cocaine, marijuana, and alcohol). Participants were also queried generally about any injection drug use at the baseline and each follow-up visit, and specifically regarding the substances they injected. For the current analysis, dichotomous variables for any injection drug in the past 6-months (measured at each visit) and past-6 month injection of NMPO and/or methamphetamine were used. Finally, a variable to distinguish new onset methamphetamine use was created to differentiate those with who began using methamphetamine at one of the follow-up visits from those with a prior history of methamphetamine use (lifetime use reported at baseline). Demographic data from the baseline interview, including age, race, gender, and years of education, were used in the models. To be consistent, opioid use disorder (OUD) (formerly opioid dependence) was assessed using DSM-IV criteria across all visits since the newer criteria were published during the follow-up period. However, since opioid dependence was assessed, that is the terminology used throughout the manuscript.

### Statistical Analyses

Given the correlated nature of the data over time, mixed effects logistic regression was used to examine longitudinal trends in recent methamphetamine use across the eight waves of data. Recent drug use variables were allowed to vary over time in the mixed effects model and estimates were exponentiated and reported as odds ratios. A forward elimination process was utilized by which substance use and demographic variables significantly (*p* < 0.05) associated with methamphetamine use over time in the simple mixed effects model were entered one at a time and changes in standard errors were observed with the addition of each new variable. The final model contains those variables that remained significantly associated with the outcome after all additional covariates were entered. The predictive margins and adjusted probabilities were calculated for recent use of methamphetamine, NMPO and heroin over time and are presented in graph form. To assess the independent correlates of past 6-month methamphetamine use at the most recent visit, simple and multivariable logistic regression was employed using the forward elimination process described above. All analyses were conducted using Stata, version 16.0 (College Station, TX).

## Results

A little less than half of the 498 NMPO in the SNAP cohort were women (45.7%) and the median age at study entry (2008–2010) was 31 years (interquartile range [IQR]: 26, 38). Consistent with the demographic composition of Appalachian Kentucky, 94.2% of NMPO were White and most participants had at least 12 years of education (IQR: 10, 12). At baseline, 84.9% of NMPOU's met DSM-IV criteria for opioid dependence and 73.5% of the sample reported injecting drugs at some point during the study timeframe, 2008–2020.

**Figure 1:**
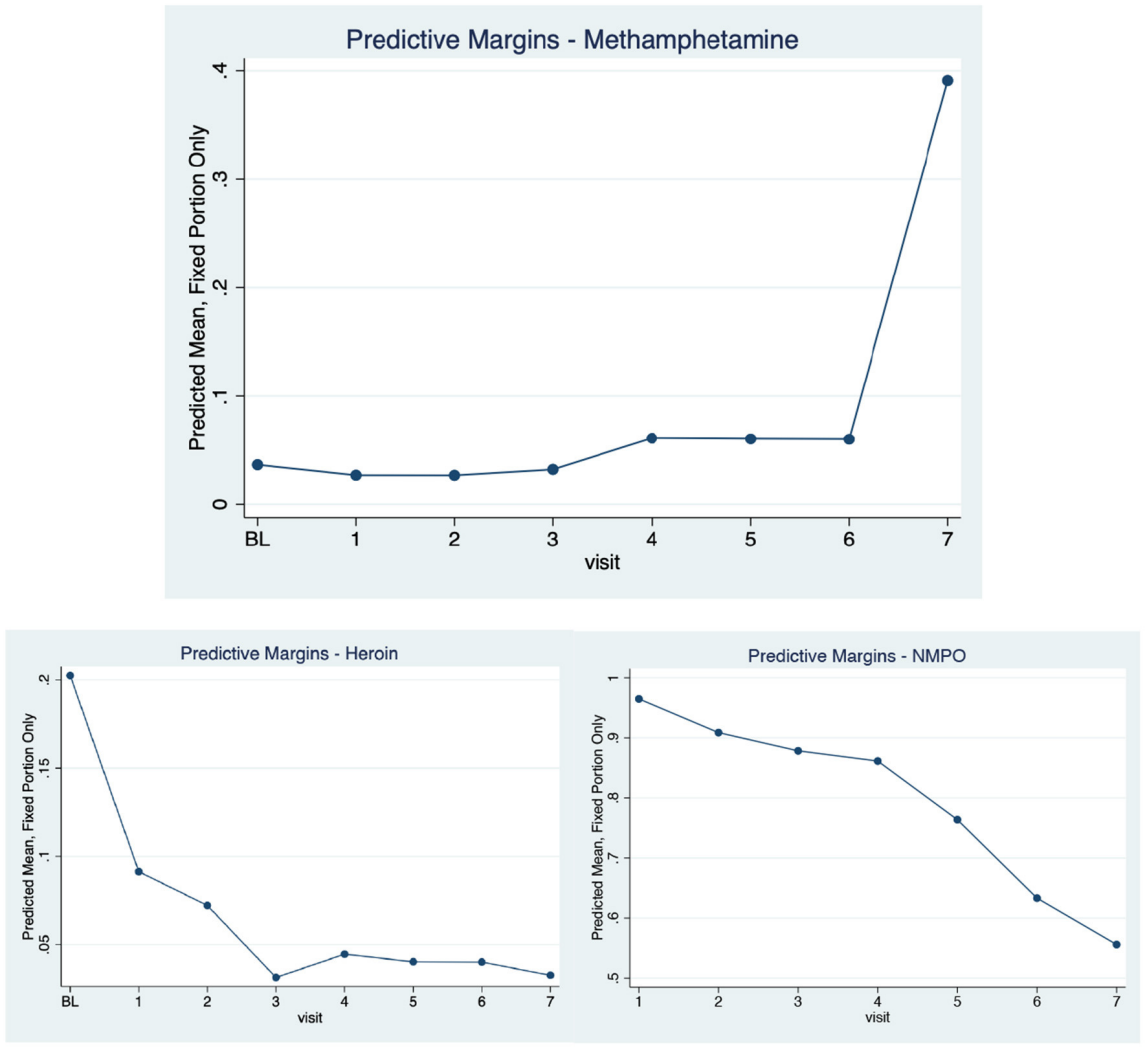
Adjusted Predicted Probabilities of Recent Methamphetamine, NMPO and Heroin Use Over Time in a Cohort of NMPO Users, 2008–2020.

There were stark changes in past 6-month (recent) use of methamphetamine over time (3,474 observations). Reports of recent use at baseline and each of the follow-up visits were as follows: 9.4, 5.6, 5.0, 5.4, 8.1, 6.8, 6.9, and 33.1%, respectively (*p* < 0.001). The increase in recent methamphetamine use was most notable at the latest follow-up visit, which was initiated in November 2017 and completed in March 2020. As seen in Table 1, recent NMPO, benzodiazepines and cocaine use were associated with increased odds of methamphetamine use over time, as was younger age. The predictive margins for the methamphetamine use model were calculated and graphed (Figure 1). The margins were also estimated for longitudinal NMPO and heroin use and the predicted probabilities are presented alongside those for methamphetamine for comparative purposes. Significant increases in the predicted probability of methamphetamine use were contrasted by statistically significant declines in both NMPO and heroin use over the past decade.

**Table 1:** Mixed effects for model of changes in methamphetamine use, 2008–2020.

**Variable**	**Adjusted odds ratio**	**95% Confidence interval**
Visit		
Baseline	1.0 (referent)	
1	0.72	0.41, 1.26
2	0.72	0.40, 1.29
3	0.87	0.49, 1.54
4	1.77	1.03, 3.03*
5	1.75	0.98, 3.13
6	1.74	0.96, 3.14
7	25.8	14.9, 44.6***
Recent (Past 6-Mo) Substance Use		
NMPO	2.52	1.61, 3.97***
Benzodiazepines	1.83	1.31, 2.57***
Cocaine	3.54	2.52, 4.97***
Lifetime Methamphetamine Use	3.07	2.06, 4.57***
Age	0.96	0.93, 0.98***
Female	1.67	1.13, 2.45**

* *p < 0.05*;

** *p < 0.01*;

*** *p < 0.001*.

**Figure 2:**
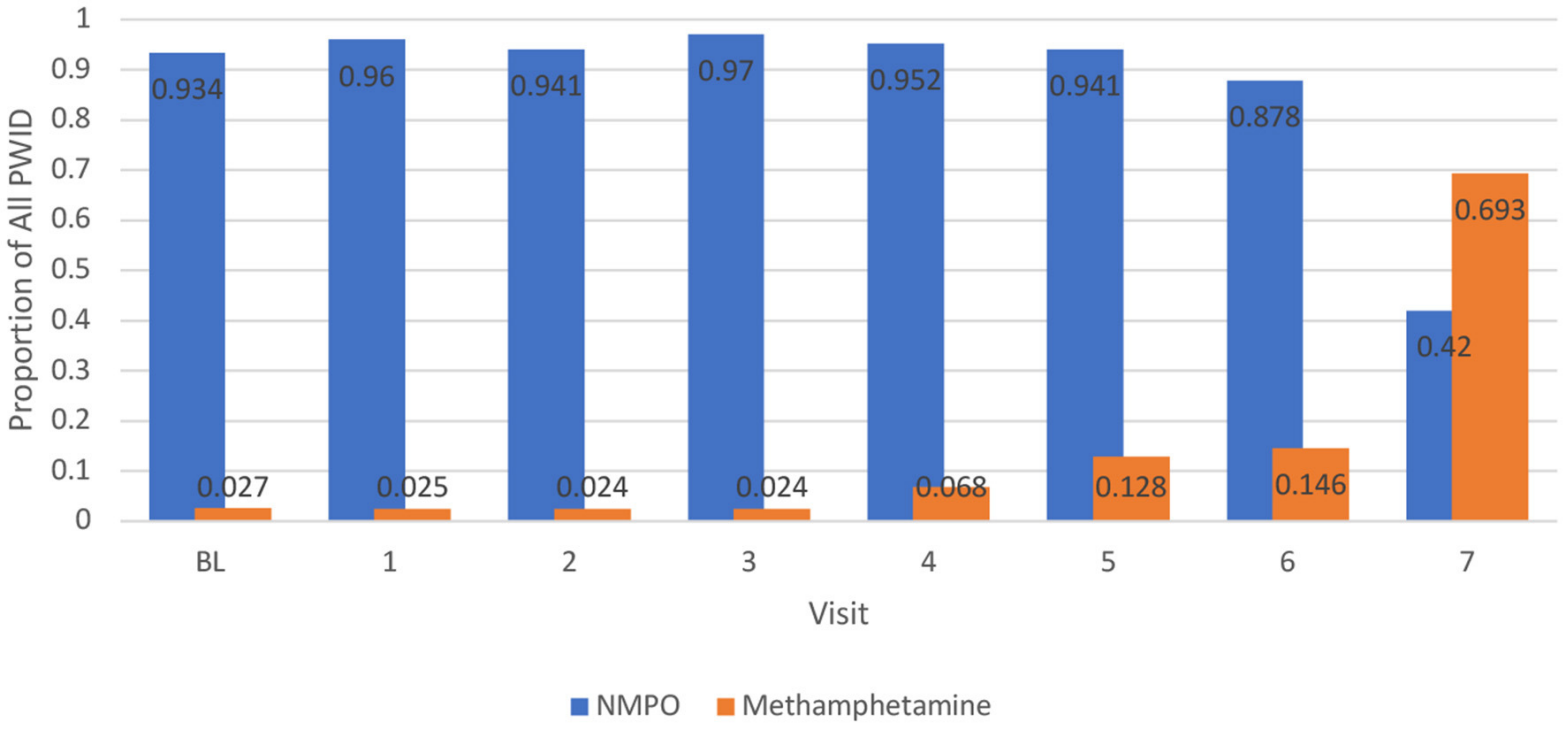
Proportion of People who Inject Drugs (PWID) Reporting Recent Injection of Methamphetamine and NMPO Over Time, 2008–2020.

A separate longitudinal model was constructed to examine recent methamphetamine injection over time since the number of observations (*n* = 1,279) was smaller for the injection-only sample of those who recently used methamphetamine. Similar to the overall model, there were significant increases in recent methamphetamine injection longitudinally (*p* < 0.001). Figure 2 compares recent injection NMPO and methamphetamine use over time. Injection of both substances is steady and dominated by NMPO, until the most recent visit, where recent injection of methamphetamine overtakes NMPO.

Finally, given the high prevalence of methamphetamine use at the most recently completed visit, a closer examination of use at this visit (*N* = 350) was undertaken. One-third (*n* = 116) of participants reported that they had used methamphetamine in the prior 6-months, and of those, 52.6% were injecting the drug. The majority of those (84.9%) had used methamphetamine in the prior 30 days and the median number of days using in the prior 30 was 10 (interquartile range: 3, 20). Among those injecting methamphetamine, the median number of days using in the past 30 was similar (10; IQR: 2, 30), but of note, the upper quartile were injecting daily. Many (38.8%, *n* = 45) of those reporting recent use were new onset users, meaning they had not reported methamphetamine use prior to the baseline interview, or methamphetamine use at any of the prior visits. The average number of new onset users in the prior visits was just under eight. Results from the cross-sectional multinomial logistic regression were not vastly different from the longitudinal model presented above. Those reporting recent methamphetamine use were significantly more likely to be younger, and using NMPO, heroin, marijuana and cocaine (Table 2) in the prior 6-months, even after adjustment for gender and pre-baseline methamphetamine use.

**Table 2:** Multivariable logistic regression examining methamphetamine use at latest visit.

**Variable**	**Adjusted odds ratio**	**95% Confidence interval**
Recent (Past 6-Mo) Substance Use		
NMPO	1.89	1.13, 3.15*
Heroin	5.89	1.57, 22.0**
Cocaine	2.73	1.36, 5.48**
Marijuana	1.77	1.07, 2.90*
Baseline Methamphetamine Use	1.28	0.77, 2.12
Age	0.96	0.93, 0.99*
Female	1.17	0.71, 1.92

* *p < 0.05*;

** *p < 0.01*.

## Discussion

As we navigate the third decade of the opioid epidemic in rural Kentucky it is clear that previous substance-related epidemics cannot adequately inform this particular crisis. The results from this study provide clear evidence for “twin epidemics” of emergent methamphetamine use among people using opioids, as this cohort comprised of NMPOUs was designed to be able to detect such trends. These “twin epidemics” are problematic on many fronts. First, and perhaps most importantly, unlike opioids, there are very few evidence-based effective treatments for MUD that could be easily implemented in rural areas, given the paucity of trained mental health professionals (30, 32) and current limitations to the real-world use of contingency management (33). So the question becomes how to leverage the strides that have been made to increase access to treatment for OUD in rural areas to also address MUD. Given the co-occurring use of NMPO and methamphetamine, there is the potential to adapt medications for OUD (MOUD) treatment protocols to address methamphetamine use for NMPOU using methamphetamine. While the evidence is not overwhelming, two studies demonstrated that use of buprenorphine reduced methamphetamine cravings (46), and those prescribed MOUD significantly reduced stimulant use while in treatment (47). A pharmacologic approach for OUD paired with one of the evidence-based psychosocial interventions for MUD (30, 48) may be ideal for this population of PWUD, but perhaps challenging to deliver in rural areas. In addition, increased availability of online interventions due to the SARS-CoV-2 pandemic may allow for penetration of evidence-based programs in rural areas. However, access to broadband internet and internet-capable devises still lags in many rural communities (49), which may ultimately limit the utility of online treatment options.

These data also suggest that once methamphetamine became readily available in the area, use significantly increased (50). At the most recent visit, there were five times the number of new onset users compared to the average at previous follow-ups. And while other areas of the U.S. who faced similar opioid crises saw this transition with heroin (3, 51), results from this cohort demonstrate that heroin use is less prevalent in this region and on the decline over time. Efforts to address the opioid epidemic may need to take into account methamphetamine use when designing and implementing interventions. And although this study was conducted among rural NMPOU in the U.S., lessons from the opioid epidemic can be used to prevent harms in areas where there are signals of problematic prescription opioid use, such as Europe and Australia (36, 38, 43, 44).

Injection of methamphetamine also significantly increased over time and overtook NMPO as the injection drug of choice among people who inject drugs (PWID) in this cohort. Given the potential for HIV and/or HCV transmission through injection and non-injection methamphetamine use (22, 23, 52), these findings only amplify the need to continue efforts to increase access to harm reduction and syringe services programs in rural areas (9). Given the association between methamphetamine use and risky sex (53), existing programs may need to also increase access to testing for sexually transmitted infections (STIs) and ensure condoms are distributed alongside injection equipment, in line with best practices for harm reduction programs (54).

### Limitations

While the potential for bias is greatly reduced in longitudinal cohort studies compared with cross-sectional designs, one concern is differential loss to follow-up (55). The mortality rate for the cohort is 10% (*n* = 50), and an additional 103 have either been removed from the study, asked to be removed, or cannot be located. Compared to those who completed the most recent follow-up, participants who were lost-to-follow-up over the course of the study were more likely to be injecting at baseline. This is not surprising given the morbidity and mortality associated with injecting drugs (56, 57). The loss of PWID over time likely did not appreciably impact the study findings, as there was sufficient power to model the injection-related outcomes. There were no differences in baseline demographics or other drug use variables between those retained and those lost-to-follow-up. If anything, the reported findings are more conservative, because additional observations for PWID would likely have led to even greater proportion of those injecting methamphetamine. Finally, measurement of the dependent variable and the majority of independent variables was reliant on self-report, which may have led to underreporting of the main outcome. However, data have shown that self-report of substance use is highly correlated with actual use (58). Despite these limitations, this represents some of the first evidence of “twin epidemics” in a longitudinal cohort of NMPOU.

In conclusion, these results provide additional evidence of the emergence of “twin epidemics” of methamphetamine and opioid use in the United States. Continued monitoring of the evolution of the opioid epidemic is essential so the harms may be understood, new treatment paradigms can be developed to address this co-occurring substance use, and appropriate prevention or intervention efforts can be implemented in regions observing the emergence of this new pattern of substance use.

## Data Availability Statement

The original contributions presented in the study are included in the article/supplementary files, further inquiries can be directed to the corresponding author.

## Ethics Statement

The studies involving human participants were reviewed and approved by Institutional Review Board, University of Kentucky. The patients/participants provided their written informed consent to participate in this study.

## Author Contributions

JH obtained funding for the study, conducted statistical analyses and drafted the manuscript. HK provided statistical and editorial support. JCS, AY, MRL, SB, and SW provided input on study hypotheses and editorial support to the drafting of the manuscript. All authors contributed to the article and approved the submitted version.

## Funding

This research was supported by grants from the National Institute on Drug Abuse (R01DA033862 and R01DA024598). NIDA had no further role in study design, in the collection, analysis, or interpretation of data, or the preparation of this manuscript.

## Author Disclaimer

The authors are solely responsible for this content, and this manuscript does not represent the official views of the NIH or NIDA.

## Conflict of Interest

The authors declare that the research was conducted in the absence of any commercial or financial relationships that could be construed as a potential conflict of interest.

## Publisher's Note

All claims expressed in this article are solely those of the authors and do not necessarily represent those of their affiliated organizations, or those of the publisher, the editors and the reviewers. Any product that may be evaluated in this article, or claim that may be made by its manufacturer, is not guaranteed or endorsed by the publisher.
